# Serum TLR9 and NF-*κ*B Biochemical Markers in Patients with Acute Pancreatitis on Admission

**DOI:** 10.1155/2020/1264714

**Published:** 2020-02-01

**Authors:** Erdal Demirtas, İlhan Korkmaz, Kıvanç Cebecioğlu, Mustafa Ayan, Esin Demirtaş, Sefa Yurtbay, Şeymanur Yıldız, Hüseyin Aydın, Lukasz Szarpak

**Affiliations:** ^1^Departments of Emergency Medicine, Sivas Cumhuriyet University Faculty of Medicine, TR-58140 Sivas, Turkey; ^2^Departments of Family Medicine, Sivas Cumhuriyet University Faculty of Medicine, TR-58140 Sivas, Turkey; ^3^Niğde Ömer Halisdemir University Training and Research Hospital, Department of Emergency Medicine, Niğde, Turkey; ^4^Departments of Biochemistry, Sivas Cumhuriyet University Faculty of Medicine, TR-58140 Sivas, Turkey; ^5^Lazarski University, Medical Faculty, Warsaw, Poland

## Abstract

**Aim:**

The aim of this study was to investigate the serum TLR9 and NF-*κ*B levels in patients for the diagnosis and prognostication of AP in the emergency department.

**Methods:**

In the current study, we looked at the TLR9 and NF-*κ*B levels in patients for the diagnosis and prognostication of AP in the emergency department.

**Results:**

Of the patients with acute pancreatitis, 22 (49%) were male and 23 (51%) were female. The mean age of the patient group was 62 years, with a range of 25–95 years. The control group consisted of 19 (43.1%) male and 25 (56.9%) female patients. The serum TLR9 and NF-*κ*B levels in patients for the diagnosis and prognostication of AP in the emergency department. *p* < 0.001 and 8.04 ± 1.76 vs. 4.76 ± 1.13; *p* < 0.001 and 8.04 ± 1.76 vs. 4.76 ± 1.13; *κ*B levels in patients for the diagnosis and prognostication of AP in the emergency department. *p* < 0.001 and 8.04 ± 1.76 vs. 4.76 ± 1.13; *κ*B levels in patients for the diagnosis and prognostication of AP in the emergency department. *p* < 0.001 and 8.04 ± 1.76 vs. 4.76 ± 1.13;

**Conclusion:**

We demonstrated that the TLR9 and NF-*κ*B pathway is activated in acute pancreatitis and increases the inflammatory process. This may help to further understand the pathogenesis of disorder, diagnosis, and clinical severity. We proposed that blockage of these inflammatory pathways may play a role in the prevention of the disease progression and development of inflammatory complications.*κ*B levels in patients for the diagnosis and prognostication of AP in the emergency department.

## 1. Introduction

Acute pancreatitis (AP) is the inflammatory disease of the pancreas characterized by damage of the acinar cells, which leads to the activation of several inflammatory cells like macrophages and granulocytes secreting several proinflammatory cytokines. Innate immunity is the first line of defense for several potential inflammatory factors. A family of toll-like receptors (TLRs) acts as a primary detector that senses a number of microbial particles and triggers innate immune responses. All TLR signaling pathways lead to the activation of the transcription factor nuclear factor-kappaB (NF-*κ*B), which controls the expression of a spectrum of inflammatory cytokine genes [1].

Toll-like receptors are transmembrane proteins that are triggered by danger-associated molecular patterns (DAMPs) from damaged tissue, resulting in the secretion of proinflammatory cytokines, chemokines, and costimulatory molecules essential for innate and adaptive responses [2]. The TLR9 signaling pathway involves the NF-*κ*B transcription factor, which is a transcription factor that determines the transcription of DNA and the initiation and development of inflammation of the diseases regulated by the inflammasomes which are regulated by NF-*κ*B. AP inflammation is occasionally associated with a systemic inflammatory response. The disease may spread to the surrounding tissues or resolve spontaneously with time. Although the disease progression is frequently (80%) mild, 20% of the patients may need critical care. Multiple organ failure generally presents within the first days and may resolve in response to the treatment [3].

Unfortunately, a “gold standard biomarker” is not established for the diagnosis of AP in the emergency department setting. Several types of laboratory, imaging, and invasive endoscopic modalities are helpful. However, there is also a need for developing new laboratory tests to assess and monitor the presence and severity of AP in order to use our healthcare resources cost-effectively while trying to follow the best clinical practice. Considering the available clinical methods used in the differential diagnosis of AP, new laboratory tests in the field of activated innate immunity may be candidate biomarkers. The aim of this study was to assess the serum TLR9 and NF-*κ*B levels in patients for the diagnosis and prognostification of AP in the emergency department.

## 2. Materials and Methods

This prospective study was conducted at the Emergency Department of Sivas Cumhuriyet University Hospital, Turkey, between February and July 2019. The Human Research Ethics Committee approved the study (protocol no: 2019-02/03), and all the patients provided written informed consent. Postpower analysis was used to evaluate the study power. During the calculations, *α* = 0.05 and the number of patients per group was 45. The difference for the variables was found by using the mean difference and common standard deviation. The power obtained using these parameters was found to be 0.999.

Patients older than 18 years who admitted with abdominal pain and were confirmed with a diagnosis of AP within 24 h of the symptom onset were included in this study (*n* = 45). The gender distribution for females and males was 51% (23) and 49% (22), respectively. The control group comprised 44 healthy persons with a mean age of 58.1 ± 8.98 years. The gender distribution for females and males was 56.8% (25) and 43.2% (19), respectively.

The diagnosis was established on the basis of acute abdominal pain, at least 3-fold elevated levels of pancreatic enzymes (amylase, lipase, and/or urine lipase), and typical radiological findings. The revised Atlanta classification was used to classify the patients' clinical severity as mild, moderate, or severe. Patients were excluded if they had evidence for chronic pancreatitis on imaging such as pancreatic duct dilation, pancreas atrophy, and presence of pancreatic calcifications. Also, patients with chronic kidney disease, pneumonia, urinary tract infection, tonsillitis, cellulitis, chronic obstructive airway disease, cirrhosis, bronchial asthma, and congestive heart failure were excluded.

### 2.1. Measurement of Blood Parameters

Mindray BC6800, China, was used for measurement of complete blood count, including hemoglobin, neutrophils, mean platelet volume, leukocytes, lymphocytes, and platelet distribution width. The C-reactive protein level was calculated with the nephelometry technique (Beckman Coulter, California, USA). Alanine aminotransferase (ALT), albumin, blood urea nitrogen (BUN), aspartate aminotransferase (AST), creatine kinase (CK), direct bilirubin, glucose, *γ*-glutamyl transferase (GGT), total bilirubin, serum creatinine, potassium, sodium, and CK-MB levels were determined with the enzymatic colorimetric method (Mindray BS2000, USA).

### 2.2. Measurement of Serum TLR9 and NF-*κ*B

Patients' blood samples were drawn, and sera fractions were separated by centrifugation (3500 rpm, 15 min, and 4 °C). They were then aliquoted and quickly kept at −80 °C (WiseCryo, South Korea). The quantitative sandwich ELISA technique was used for the determination of serum TLR9 and NF-*κ*B (Elabscience Biotechnology Co., Ltd, China). Tests were carried out according to the manufacturer's recommendations.

### 2.3. Statistical Analysis

IBM SPSS Statistics for Windows version 23.0 (IBM, Armonk, NY, USA) was used for statistical analysis. The Kolmogorov–Smirnov test was used as a test for normality of continuous data, and the results were expressed as mean ± standard deviation (SD) or median with the 25th and 75th percentiles. The nonparametric Mann–Whitney *U* test was used to compare the medians between the two groups. Receiver operating characteristic (ROC) analysis was used to determine the diagnostic efficiency of TLR9, and NF-*κ*B and the areas under the curve (AUCs) were compared. *p* < 0.05 was considered statistically significant. Logistic regression analysis was used in order to determine the factors affecting acute pancreatitis.

## 3. Results

Of the patients with acute pancreatitis, 22 (49%) were male and 23 (51%) were female. The mean age of the patient group was 62 years, with a range of 25–95 years. The control group consisted of 19 (43.1%) male and 25 (56.9%) female patients. The mean age of the control group was 63 years, with a range of 21–91 years. There was no significant difference between the patients and the control group regarding the age and gender of the study groups (*p* > 0.05).

Table 1 presents the CBC and biochemistry laboratory tests with acute pancreatitis of the admission.

**Figure 1:**
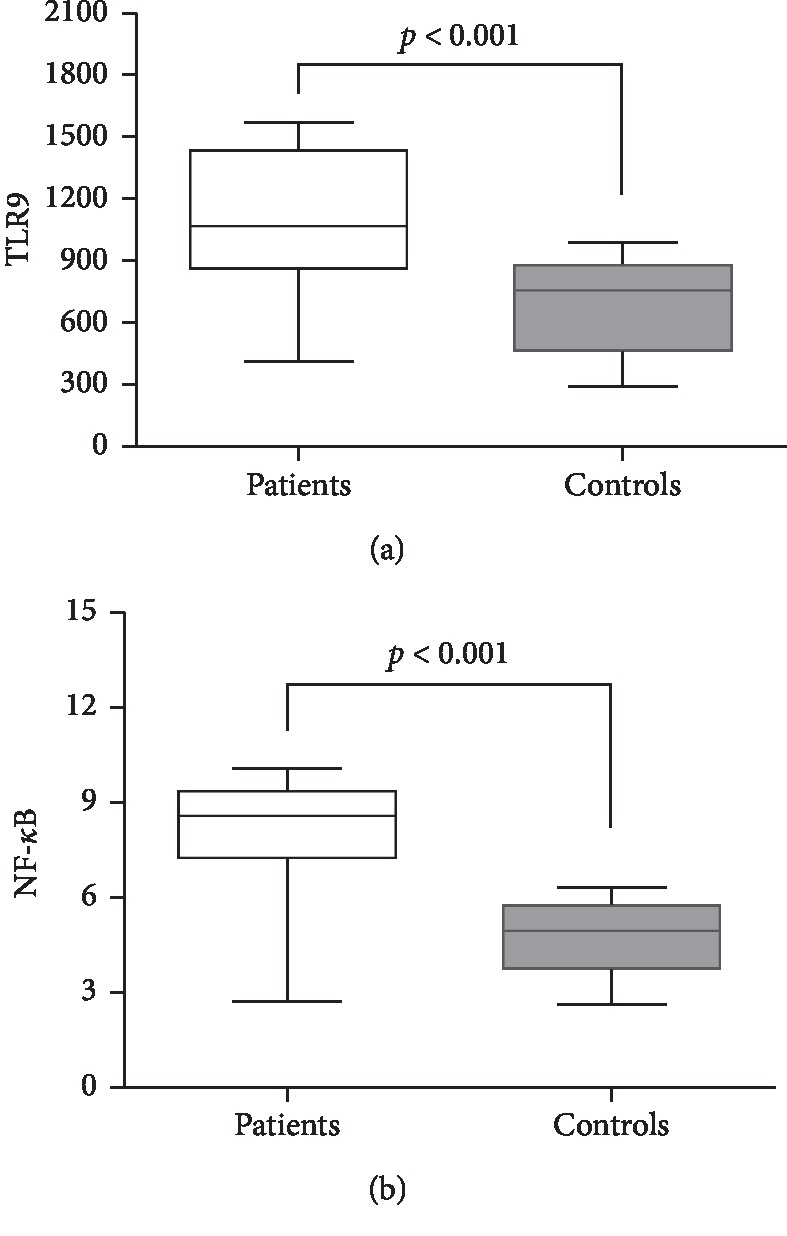
The comparison of serum TLR9 and NF-*κ*B values of patients with acute pancreatitis and control groups. The serum TLR9 values (a) (1104.44 ± 339.20 vs. 702.08 ± 203.94; (*p* < 0.001)) and NF-*κ*B values (b) (8.04 ± 1.76 vs. 4.76 ± 1.13; (*p* < 0.001)).

In patients with acute pancreatitis, the serum TLR9 and NF-*κ*B values were significantly higher than those of the control group [1104.44 ± 339.20 vs. 702.08 ± 203.94; *p* < 0.001 and 8.04 ± 1.76 vs. 4.76 ± 1.13; *p* < 0.001, respectively].  Figure 1 and Table 2 show the comparison of serum TLR9 and NF-*κ*B values of patients with acute pancreatitis and control groups.

As shown in Table 3, serum TLR9 and NF *κ*B values in the patients with acute pancreatitis, there were no significant differences between the mild-moderate and severe groups. (*p*=0.866 and *p*=0.604, respectively).

**Figure 2:**
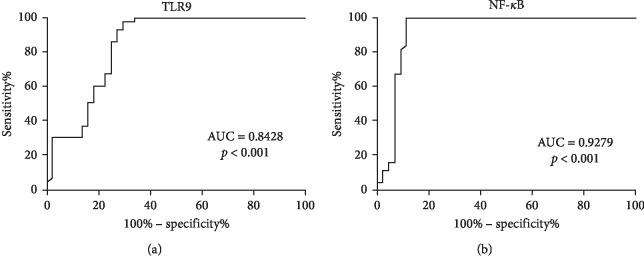
ROC analyses demonstrated the performances of the TLR9 and NF-*κ*B in the prediction of acute pancreatitis. TLR9 had a cutoff value of 950.4, with a sensitivity of 73% and specificity of 93% (*p* < 0.001) (a), and NF-*κ*B had a cutoff value of 6.32, with a sensitivity of 89% and specificity of 100% (*p* < 0.001) (b).

As presented in Figure 2, ROC analyses demonstrated the performances of the TLR9 and NF-*κ*B in the prediction of acute pancreatitis. We found that the TLR9 and NF-*κ*B had a significant discriminative ability, while the cutoff value for TLR9 was 950.4, with a sensitivity of 73% and specificity of 93% (*p* < 0.001), and the cutoff value for NF-*κ*B was 6.32, with a sensitivity of 89% and specificity of 100% (*p* < 0.001). As shown in Table 4, while TLR9 increased 1.005 times, NF-*κ*B increased 3.52 times the diagnosis of pancreatitis according the logistic regression analysis.

## 4. Discussion

AP is a common diagnosis among the patients who were admitted to the emergency department with abdominal pain and represented both a diagnostic and therapeutic challenge. In this study, we tested the predictive value of TLR9 and NF-*κ*B for the diagnosis and prognostification of acute pancreatitis, where we compared the TLR9 and NF-*κ*B among the patient and control groups. The mean values among the patient groups were significantly higher (*p* < 0.001 for TLR9 and NF-*κ*B), and the logistic regression revealed the diagnostic value for pancreatitis (*p* < 0.001). It is known that NF-*κ*B and TLR9 induce inflammatory response and systemic reactions in acute pancreatitis. However, the TLR9 and NF-*κ*B levels of the mild-moderate and severe groups did not differ from each other significantly to differentiate the severity of the two groups.

AP may be evaluated in two phases. The first phase includes regional inflammation and necrosis of the pancreas that ends up with the generation of several inflammatory mediators. The second phase involves the systematic reaction induced by the overall transmission of those inflammatory cytokines and is characterized by systemic inflammatory reaction syndrome.

Aparna et al. [4] studied the early interaction between human pancreatic acinar injury with inflammatory cytokines in the pathogenesis of AP using the pancreatic tissue acquired from patients undergoing pancreatic resection. According to their study, they proposed that bile acid exposure to the acini is the initial impact that causes acinar injury and intrapancreatic secretion of proinflammatory cytokines by the acinar cells. The released cytokines attract and activate peripheral blood mononuclear cells (PBMCs) within the pancreas. These activated PBMCs pass into the systemic circulation and additionally release cytokines acting as the second impact to the pancreatic acinar cells and also cause early systemic inflammatory response syndrome and multiorgan dysfunction.

Hoque et al. studied the inflammasomes (ASC, NLRP3, and caspase-1), toll-like receptor 9 (TLR9), and the purinergic receptor P2X7 levels in cerulein stimulation-induced acute pancreatitis mice model. They showed that caspase-1, ASC, and NLPR3 were necessary for inflammation in acute pancreatitis. Genetic deficiency of TLR9 alleviates pancreatic edema, inflammation, and pro-IL-1*β* expression in pancreatitis. Pretreatment with the TLR9 antagonist IRS954 alleviates pancreatic edema, inflammatory infiltrate, and apoptosis [5].

In the current literature, no studies existed investigating the diagnostic value of TLR9 and NF-*κ*B in AP. The traditional diagnostic biomarkers for acute pancreatitis are serum amylase and lipase levels. At least three-time increased upper normal serum amylase level with the clinical complaints is used for the diagnosis. Serum amylase level rises quickly within the first 12 hours after the onset of symptoms and returns to normal within three to five days. However, due to the delayed admission or exocrine insufficiency, serum amylase activities may be normal in 19–32% cases at the time of hospital admission [6–8].

Zeng et al. [9] showed that TLR9 was expressed in cerulein-induced pancreatitis in rats. They found that TLR9 mRNA upregulation initiated within 30 minutes and achieved the culminating point in the 1-hour after the induction of the pancreas with cerulein. The TLR9 mRNA turned to the baseline levels within 24 hours. Sun et al. [10] determined the levels of TLR9 protein expression in rats with acute pancreatitis and compared them with a control group. The TLR9 rate was upregulated in the acute pancreatitis rats during 3, 6, and 12 h groups when compared with the control group. The peak upregulation of TLR9 expression levels was in the 6th hour.

NF-*κ*B is a cytokine that is produced by many transcription factors, and its production is activated within 30 minutes of the initiation of acute pancreatitis [11]. Satoh et al. studied 45 patients who were admitted with acute pancreatitis and compared the NF-*κ*B rate expression in blood mononuclear cells at admission and 14 days later with the control group. They found that NF-*κ*B levels among the pancreatitis group were higher than the control group at the admission time, and the patients with serious complications had a persistently high NF-*κ*B level during the hospitalization time [12].

The results of our study were consistent with this aforementioned literature. In our study, TLR9 was significantly higher in the patient group compared with the control group. The activated TLR9 pathway in acute pancreatitis also induces NF-*κ*B, which activates the inflammation of the pancreas and the surrounding tissues. However, further investigations are needed to investigate the presence of other factors that activate NF-*κ*B. The sensitivity of TLR9 values that we measured in patients with acute pancreatitis was close to the currently used diagnostic biomarkers. We also suggest that analysis of admission NF-*κ*B has a diagnostic value among suspected acute pancreatitis patients with relatively high sensitivity and specificity.

Different approaches have been utilized to evaluate the severity of acute pancreatitis and predict prognosis. Several biological markers have also been tested for this purpose. Genetic markers are being evaluated and have not yet been used widely. The level of serum amylase or lipase does not discriminate whether the disease is mild-moderate or severe, and monitoring levels serially during the course of hospitalization does not present insight into the prognosis [13]. One of the early experimental studies for the differentiation of acute pancreatitis severity according to the biochemical biomarkers was made by Leser et al., they observed the interleukin-6 and CRP during the hospitalization time. They found that the IL-6 serum concentrations had sensitivity, specificity, positive predictive value, and negative predictive value for severe or lethal pancreatitis as 80%, 92%, 91%, and 82%, respectively. The parameters were also analyzed for CRP and determined as 83%, 62%, 67%, and 79%, respectively [14]. Huang et al. evaluated the correlation between the level of NF-*κ*B and the severity of pancreatitis. They found that p65 transgenic mice induced with cerulein to create acute pancreatitis had higher levels of NF-*κ*B activities in acinar cells, a wider area of inflammation, and severe outcome [15]. Chen et al. evaluated the activation effect of NF-*κ*B on local injury or systemic inflammatory response in acute pancreatitis mice. They supported that NF-*κ*B activity in pancreatic acinar cells stimulates the inflammatory response during acute pancreatitis and therapeutic intervention on the NF-*κ*B/IKB system can be important to reduce the clinical severity of acute pancreatitis [16]. The relation between NF-*κ*B and the clinical severity pancreatitis is also showed by different studies [15, 17].

Currently, there are no approved therapies for acute pancreatitis. On the contrary, recent studies have shown that decursin blocking the NF-*κ*B pathway is a candidate for the treatment of many inflammatory diseases [18]. There are also some studies that show the protective effect of NF-*κ*B inhibition by different ways in acute pancreatitis for the prognosis [19–21]. Satoh et al. determined that the fatal outcome rate was lower among the rats with taurocholate-induced pancreatitis when it inhibited NF-*κ*B with pyrrolidine dithiocarbamate. Ethridge et al. [21] evaluated the protective effect of a novel peptide that binds the NF-*κ*B essential modifier binding domain and inhibits the activity of NF-*κ*B. Treatment with the NBD peptide decreased the severity and complication rates.

Our study had several limitations. First, the sample size was relatively small, that is why the results of the study should be interpreted with caution. Second, a single TLR9 and NF-*κ*B measurement may not reflect the long-term course.

## 5. Conclusion

In our study, we demonstrated that the TLR9 and NF-*κ*B pathway is activated in acute pancreatitis, which is an inflammatory process. This may help to further understand the pathogenesis of disorder and development of new treatment modalities considering functions of the TLR9 and NF-*κ*B signaling. We proposed that blockage of these inflammatory pathways may play a role in the prevention of the disease progression and development of inflammatory complications.

## Figures and Tables

**Table 1:** Laboratory result of patients at the admission.

Laboratory	Admission (median 25–75)
WBC	11.70 (7.85–14.75)
Neutrophil count	85.00 (76.1–90.2)
Lymphocyte count	9.70 (5.5–17.5)
Monocyte count	4.95 (3.8–5.85)
Hemoglobin, g/dL	13.70 (12.2–15.5)
Hematocrit, %	40.50 (36.5–44.5)
MCV, fL	87.90 (84.7–92)
MCHC, fL	33.90 (33.15–34.55)
Platelet count, 10^9^	217.00 (177–285)
MPV	9.40 (8.7–10.2)
BUN, mg/dL	14.46 (11.7–18.86)
Serum creatinine, mg/dL	0.80 (0.62–0.95)
Albumin, g/dL	3.80 (3.45–4.40)
Amylase, U/L	867.00 (299–1466)
Lipase, U/L	1363.00 (548–1621)
Urine amylase, U/L	3119.00 (1487–3814)
ALP, U/L	127.50 (87–197)
ALT, U/L	128.00 (33–266)
AST, U/L	123.00 (38–299)
LDH, U/L	334.00 (232–506)
GGT, U/L	220.00 (60–422)
Calcium, mmol/L	8.87 (8.59–9.09)
Glucose, mg/dL	116 (98–144)
CRP, mg/dL	21.9 (7.63–144)

**Table 2:** Comparison of TLR9 and NF-*κ*B between patient and control groups.

Biomarkers	Group		*p*	Postpower
	Patients (*n* = 45)	Controls (*n* = 44)		
TLR9	1104.44 ± 339.20	702.08 ± 203.94	<0.001	0.999
NF-*κ*B	8.04 ± 1.76	4.76 ± 1.13	<0.001	0.999

Data are given as mean ± SD.

**Table 3:** TLR9 and NF-*κ*B levels according to the severity of pancreatitis.

Biomarkers	Mild-moderate (*N* = 27)	Severe (*N* = 18)	*p* value
TLR9	1038.2 (809.0–1264)	8.55 (6.38–8.72)	0.866
NF-*κ*B	1186.5 (545.3–1213.5)	8.62 (7.00–8.70)	0.604

TLR9, toll-like receptor 9; NF-*κ*B, nuclear factor-kappaB. Data were expressed as median (25%–75% interquartile range) and were analyzed with the Mann–Whitney *U* test.

**Table 4:** Evaluation of TLR9 and NF-*κ*B variables as risk factors affecting acute pancreatitis.

Biomarkers	OR (95% CI)	*p*
TLR9	1.005 (1.003–1.007)	<0.001
NF-*κ*B	3.524 (2.126–5.841)	<0.001

OR: odds ratio; CI: confidence interval.

## Data Availability

All the authors declare that the data used to support the findings of this study are available.
